# Rest–Activity Disturbances Correlate with Core Features in Dementia with Lewy Bodies

**DOI:** 10.1002/mdc3.70052

**Published:** 2025-03-25

**Authors:** Jack Anderson, Nicholas K.H. Chiu, Jonathon E. Pye, Maria Comas‐Soberats, Aaron Lam, Ronald R. Grunstein, Simon J.G. Lewis, Elie Matar

**Affiliations:** ^1^ Faculty of Medicine and Health, Central Clinical School The University of Sydney Sydney New South Wales Australia; ^2^ Centre for Integrated Research and Understanding of Sleep (CIRUS) Woolcock Institute of Medical Research Sydney New South Wales Australia; ^3^ Department of Psychiatry Pamela Youde Nethersole Eastern Hospital Hong Kong, SAR China; ^4^ Department of Medicine and Therapeutics, Faculty of Medicine, Margaret K.L. Cheung Research Centre for Management of Parkinsonism The Chinese University of Hong Kong Hong Kong, SAR China; ^5^ Healthy Brain Aging Program, Brain and Mind Centre The University of Sydney Sydney New South Wales Australia; ^6^ Faculty of Science, School of Psychology The University of Sydney Sydney New South Wales Australia; ^7^ Charles Perkins Centre The University of Sydney Sydney New South Wales Australia; ^8^ Faculty of Medicine, Health and Human Sciences, Macquarie Medical School Macquarie University Sydney New South Wales Australia; ^9^ RPA‐Charles Perkins Centre Clinic Royal Prince Alfred Hospital Sydney New South Wales Australia; ^10^ Faculty of Medicine, Health and Human Sciences, Parkinson's Disease Research Centre, Macquarie Medical School Macquarie University Sydney New South Wales Australia; ^11^ Department of Neurology Royal Prince Alfred Hospital Sydney New South Wales Australia

**Keywords:** dementia with Lewy bodies, DLB, actigraphy, sleep, nonparametric

## Abstract

**Background:**

Sleep–wake disturbances are a clinically important and poorly studied feature of dementia with Lewy bodies (DLB) due to the challenges of in‐laboratory polysomnography in this population.

**Objectives:**

To compare rest–activity rhythms in DLB, Parkinson's disease (PD), and age‐matched controls using home‐based wrist actigraphy and examine their relationship with core clinical DLB features.

**Methods:**

Eleven DLB patients, 12 PD patients, and 11 age‐matched controls underwent clinical assessment. Actigraphy data were obtained over 14 days and analyzed using nonparametric methods.

**Results:**

DLB patients demonstrated higher rest–activity rhythm fragmentation (*P* = 0.002) than controls and lower circadian amplitude (*P* = 0.011) than both PD and controls. Instability of rest–activity rhythm was positively correlated with hallucinations (*P* = 0.009) and cognitive fluctuations (*P* = 0.016) in DLB, and reduced daytime activity correlated with severity of motor parkinsonism (*P* = 0.013). No such correlations were observed in PD.

**Conclusions:**

Actigraphy detects distinct rest–activity rhythm disruptions in DLB, differentiating it from PD and controls. These measures are associated with the severity of core clinical features in DLB.

Dementia with Lewy bodies (DLB) is the second most common cause of neurodegenerative dementia in older adults, yet it remains widely underdetected and often misdiagnosed.^1^ Sleep–wake and circadian disturbances are commonly reported^2^ and have been shown to be associated with neuropsychiatric symptoms, such as cognitive fluctuations and visual hallucinations.^3^ These neuropsychiatric features, along with parkinsonism and rapid eye movement sleep behavior disorder (RBD), constitute the core clinical features of DLB and collectively contribute to high rates of caregiver burden and poor quality of life.^4^ Despite the importance of these troublesome symptoms, biomarkers are lacking, with assessment limited to subjective interview and questionnaires.

Polysomnography (PSG) is the gold standard for diagnosing and monitoring sleep disturbances but is limited by accessibility, the requirement of extensive time investment from staff and patients, and the inability to measure activity during the day and over an extended period of time. Wrist actigraphy is an easy‐to‐use, set‐and‐forget, small device that offers a cost‐effective at‐home alternative for capturing daily variations in activity over time. Actigraphy‐derived sleep measures have been shown to correlate well with PSG in Alzheimer's dementia (AD)^5^ and Parkinson's disease (PD).^6^ Furthermore, analysis methods have evolved with the validation of nonparametric actigraphy measures, which quantify sleep stability, sleep fragmentation, nighttime arousals, daytime napping, and the amplitude of circadian rhythm through continuous monitoring, which cannot be practically derived from PSG.^7^ Changes in these outcomes have been associated with an increased risk of developing AD and faster progression from mild cognitive impairment to AD,^8^ but studies in DLB patients are limited.

In this study, we investigated the differences in rest–activity and circadian rhythms measured using actigraphy between patients with DLB, those with PD, and age‐matched controls. In addition to demonstrating its utility in DLB, we aimed to explore the association between rest–activity and circadian disturbances with cognitive, motor, and neuropsychiatric clinical variables in DLB. We hypothesized that patients with DLB would have a higher volume and severity of rest–activity disturbances than PD patients and controls. We also expect these disturbances will correlate with a higher burden of cognitive, motor, and neuropsychiatric symptoms.

## Patients and Methods

### Patients

Patients diagnosed with DLB^9^ and PD^10^ within 5 years of diagnosis and age‐matched controls without cognitive impairment on formal neuropsychometric testing were recruited from the Brain and Mind Centre, University of Sydney. Participants were assessed by a specialist neurologist, and controls were screened for any sleep, psychiatric, or neurological disorders. No patients were taking serotonin, noradrenergic reuptake inhibitors, or antipsychotics at the time of the study. PD patients were assessed on their usual dopaminergic medications.

### Actigraphy

Eligible patients wore an Actiwatch Spectrum Plus (Philips Respironics, Bend, OR, USA) actigraphy watch over a 14‐day period, placed on the nondominant wrist. A sleep diary was completed concurrently with support from a caregiver. Data were extracted using *Actiware* (version 6) software, and major rest intervals were manually scored by trained researchers and sleep technicians, who were blinded to group assignment, with cross‐validation from the carer‐supported sleep diary. Periods that reflected the removal of the watch were excluded from the analysis. Nonparametric measures were calculated in *RStudio* using the nparACT package.^7^

**FIG. 1:**
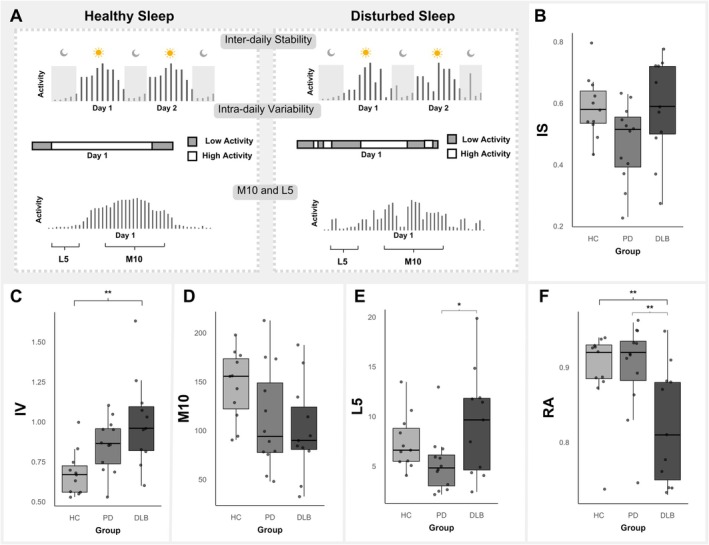
Box plots of nonparametric actigraphy variables between DLB patients, PD patients, and age‐matched controls. **Post hoc comparison is significant at the 0.01 level. *Post hoc comparison is significant at the 0.05 level. (**A**) A conceptual graphic illustration of the nonparametric actigraphy variables inter‐daily stability, intra‐daily variability, M10, and L5. The illustration is a visual representation of how each nonparametric actigraphy variable may differentiate between healthy and disturbed sleep–wake activity. Inter‐daily stability reflects the stability of rest–activity rhythm, intra‐daily variability returns the fragmentation of the rest–activity rhythm, M10 is the average activity during the most active 10 hr of each day, and L5 is the average daily activity during the least active 5 hr of the day. Relative amplitude is calculated using M10 and L5 using the following equation: RA = (M10 – L5)/(M10 + L5). (**B**–**F**) Individual box plots of group differences in nonparametric actigraphy variables between DLB, PD, and age‐matched control participants. HC, healthy controls; IS, inter‐daily stability; IV, intra‐daily variability; L5, average daily activity during least active 5 hr of each day; M10, average activity during the most active 10 hr of each day; PD, Parkinson's disease; RA, relative amplitude; DLB, dementia with Lewy bodies.

Actigraphy variables assessed included total sleep time (TST), time in bed (TIB), and percentage of wake time (%WT). Nonparametric variables included inter‐daily stability (IS), intra‐daily variability (IV), average daily activity during least‐active 5 hr of each day (L5), average activity during the most active 10 hr of each day (M10), and relative amplitude (RA).^11^ Detailed definitions of all actigraphy variables are presented in Table S1 and are summarized in Figure 1A.

### Questionnaires and Assessments

All patients undertook the following: Mini‐Mental State Examination (MMSE) and the Hospital Anxiety and Depression Scale (HADS). Patients with PD or DLB completed additional tests: REM Sleep Behavior Disorder Screening Questionnaire (RBDSQ); Clinician Assessment of Fluctuation Scale total score; Psychosis and Hallucinations Questionnaire Part I; Movement Disorders Society Unified Parkinson's Disease Rating Scale (MDS‐UPDRS), Parts III and IV; and the Scales for Outcomes in Parkinson's Disease‐Daytime Sleep (SCOPA‐S Day).

### Statistical Analysis

*RStudio* ^12^ was used to perform statistical analyses. To compare demographic, questionnaire, and actigraphy measures between healthy controls, PD, and DLB, we used permutation‐based (10,000 permutations) general linear models and χ^2^ tests for categorical data. For nonnormal distributions, correlation coefficients used Spearman's rho. All tests were 2‐tailed, with α set at 0.05. Due to the sample size and exploratory nature of the study, correlations were presented without controlling for multiple comparisons.^13^, ^14^

## Results

Eleven DLB patients (1 woman), 12 PD patients (3 women), and 11 age‐matched controls (4 women) completed all study procedures.

DLB patients were older than PD patients but had similar length of disease duration and were less medicated. As expected, DLB patients had significantly worse cognitive scores (MMSE) compared to PD patients and controls. They also had increased motor symptom severity (UPDRS, Part III) and reported increased RBD symptoms (RBDSQ) and daytime sleepiness (SCOPA‐S) than PD patients and higher depression scores (HADS‐D) compared to controls.

Actigraphy measures revealed a greater total TIB and %WT in DLB compared to PD. DLB patients had significantly higher IV than controls and lower RA than both comparative groups. See Table 1.

**TABLE 1:** Descriptive, neuropsychiatric, motor, cognitive, and sleep data for DLB patients, PD patients, and controls

	Age‐matched controls	PD	DLB	*P*‐value	Post hoc
Demographics					
n	11	12	11	–	
Age	67.18 (8.77)	62.41 (5.49)	74.50 (7.43)	**0.001**	DLB > PD
Male (%)^a^	7 (63.64)	8 (66.67)	10 (90.91)	0.279	
Disease duration (y)	N/A	2.25 (1.60)	2.10 (1.58)	0.869	
Hoehn and Yahr	N/A	1.55 (0.52)	1.67 (0.87)	0.753	
DDE	N/A	43.60 (101.00)	273.00 (285.00)	**0.006**	
MDS‐UPDRS, Part III	N/A	20.42 (9.61)	31.55 (12.04)	**0.022**	
MDS‐UPDRS, Part IV	N/A	0.75 (2.05)	0.00 (0.00)	0.316	
Questionnaires and assessments					
MMSE	29.18 (1.25)	29.10 (1.51)	22.89 (7.22)	**0.002**	DLB < HC, DLB < PD
HADS‐D	2.09 (1.25)	2.92 (2.90)	6.60 (5.34)	**0.001**	**DLB > HC**
HADS‐A	3.73 (2.72)	4.00 (3.46)	4.10 (2.73)	0.950	
RBDSQ	N/A	4.33 (3.65)	7.55 (3.58)	**0.044**	
PsycH‐Q‐Section I	N/A	1.17 (2.33)	3.22 (4.18)	0.174	
SCOPA‐S (Day)	N/A	3.17 (1.75)	6.36 (4.22)	**0.029**	
CAF	N/A	N/A	4.86 (3.80)	N/A	
Actigraphy measures					
TIB	503.61 (52.40)	473.23 (42.72)	552.27 (67.74)	**0.006**	**DLB > PD**
TST	451.89 (52.77)	441.03 (40.57)	473.02 (63.41)	0.342	
%WT	10.85 (3.46)	6.77 (2.01)	14.03 (7.79)	**0.008**	**DLB > PD**
IS	0.59 (0.10)	0.47 (0.13)	0.58 (0.16)	0.053	
IV	0.68 (0.14)	0.86 (0.17)	1.00 (0.28)	**0.002**	**DLB > HC**
M10	146.29 (35.49)	112.02 (52.73)	100.97 (47.70)	0.081	
L5	7.46 (2.77)	5.25 (2.93)	9.34 (5.31)	**0.049**	**DLB > PD**
RA	0.90 (0.06)	0.90 (0.06)	0.82 (0.08)	**0.010**	**DLB < HC, DLB < PD**

Data expressed as mean (standard deviation). Values in bold font denote statistical significance at the *P* < 0.05 level.

Abbreviations: DLB, dementia with Lewy bodies; PD, Parkinson's disease; DDE, dopamine dose equivalent; MDS‐UPDRS‐III, Movement Disorders Society Unified Parkinson's Disease Rating Scale, Part III; MDS‐UPDRS‐IV, Movement Disorders Society Unified Parkinson's Disease Rating Scale, Part IV; MMSE, Mini‐Mental State Examination; HC, healthy control; HADS‐D, Hospital Anxiety and Depression Scale‐Depression; HADS‐A, Hospital Anxiety and Depression Scale‐Anxiety; RBDSQ, REM Sleep Behavior Disorder Screening Questionnaire; PsycH‐Q, Psychosis and Hallucinations Questionnaire; SCOPA‐S (Day), Scales for Outcomes in Parkinson's Disease‐Daytime Sleep; CAF, Clinician Assessment of Fluctuation Scale; TIB, time in bed; TST, total sleep time; %WT, percentage of wake time; IS, inter‐daily stability; IV, intra‐daily variability; L5, average daily activity during least active 5 hr of each day; M10, average activity during the most active 10 hr of each day; N/A, not assessed; RA, relative amplitude.

^a^ χ^2^ test.

We next explored the associations between nonparametric actigraphy measures and core clinical features in patients with DLB. We found lower IS to be strongly correlated with increased severity of cognitive fluctuations (r = −0.701, *P* = 0.016) and hallucinations (r = −0.801, *P* = 0.009) and moderately correlated with subjective daytime sleepiness (r = −0.659, *P* = 0.027). Lower mean activity during the most active 10 hr of the day (M10) was strongly correlated with increased motor symptom severity (UPDRS, Part III) (r = −0.720, *P* = 0.013). There were no significant correlations between actigraphy measures and disease duration (IS: r = −0.206, *P* = 0.545; IV: r = 0.375, *P* = 0.256; M10: r = 0.264, *P* = 0.433; L5: r = 0.539, *P* = 0.087; RA: r = −0.530, *P* = 0.093), cognition (MMSE) (IS: r = 0.207, *P* = 0.594; IV: r = 0.00, *P* = 1.00; M10: r = 0.390, *P* = 0.300; L5: r = −0.454, *P* = 0.220; RA: r = −0.218, *P* = 0.572), or motor symptom complications (UPDRS‐IV) (IS: r = 0.00, *P* = 1.00; IV: r = 0.00, *P* = 1.00; M10: r = 0.00, *P* = 1.00; L5: r = 0.00, *P* = 1.00; RA: r = 0.00, *P* = 1.00). The full correlation details are presented in Table S2.

These associations between nonparametric actigraphy measures and clinical features were also explored in patients with PD. However, no significant correlations were found. See Table S3.

## Discussion

In this study, we demonstrate that wrist actigraphy measures effectively capture variations in sleep and rest–activity patterns when DLB patients, PD patients, and age‐matched controls are compared. Moreover, specific disturbances in rest–activity and circadian rhythms, extracted from nonparametric actigraphy measures, strongly correlated with core neuropsychiatric and motor features of DLB.

First, our findings highlight unique rest–activity and circadian disruptions in DLB compared to PD patients and healthy older adults. DLB patients exhibited longer TIB, higher wake percentage, and increased activity during the least active hours compared to PD patients, alongside greater rhythm fragmentation and lower circadian amplitude than both PD patients and controls. These heightened rest–activity disturbances in DLB have important clinical implications. Sleep disturbances can increase caregiver burden and distress,^15^ and may contribute to negative health outcomes through the known relationship of sleep, health, and cognition.^16^ Further research into the neurobiological underpinnings of rest–activity and circadian rhythms in DLB is needed. Sleep fragmentation has been found to correlate with a greater Lewy body pathology burden,^17^ whereas TST and sleep activity count measured by actigraphy has been associated with thalamic dysfunction in DLB patients.^18^ AD pathology is observed in up to 60% of patients with DLB and is an important part of the pathophysiology underlying DLB^19^, ^20^, ^21^ and has also been shown to influence rest–activity patterns.^22^, ^23^

Importantly, this study is the first to investigate nonparametric actigraphy measures in DLB and their associations with core clinical features. Here, we found that disrupted entrainment to the 24‐hr day–night cycle correlated with the increased burden of cognitive fluctuations and hallucinations. Our data suggest that disruption of the circadian rhythms together with the other components of the sleep–wake circuitry may play a role in the pathophysiology of neuropsychiatric symptoms of DLB. Tracking rest‐actigraphy rhythm fragmentation in DLB patients may predict which patients are more likely to experience cognitive fluctuations and hallucinations, symptoms that are associated with both an increased likelihood of hospitalization and/or institutionalization.^4^ Interestingly, no core DLB features were associated with activity during the least active 5 hr of the day or with circadian rhythm amplitude, perhaps suggesting a specific role of circadian dysfunction in these neuropsychiatric symptoms. Disease duration in DLB did not correlate with any nonparametric actigraphy measures, although the relatively short average disease duration in this study may have influenced these findings.

In addition to neuropsychiatric symptoms, our study found reduced daytime activity (M10) correlated with increased motor symptom severity. Whereas not seen in our cohort of early PD with shorter disease duration, associations between flattened diurnal motor activity and disease progression,^24^ along with disease severity, have been found in more advanced PD studies.^25^ Our study demonstrates that this relationship likely extends to DLB. Therefore, tracking circadian rhythm amplitude and reduced daytime activity via actigraphy may serve as a cost‐effective and simple‐to‐use tool to track motor impairment in DLB.

Finally, this study also demonstrates the feasibility of collecting wrist actigraphy assessments from DLB patients. Although PSG is generally considered the “gold standard,” the decreased cost, increased evaluation period, and elimination of the first‐night effect associated with PSG^26^ make wrist actigraphy an important tool in both clinical and research settings.

There are several limitations that should be noted. First, with wrist actigraphy, sleep is inferred by movement measures, and confirmatory electrophysiological information was not captured. Second, whereas nonparametric actigraphy methods have been successfully employed in clinical populations, new exploratory methods of actigraphy analysis, such as state‐transition analysis,^27^ may be of benefit in understanding state changes as seen in cognitive fluctuations, for example. Although DLB patients have cognitive and motor impairments, actigraphy may overestimate sleep due to reduced movement. To account for this, we included a PD comparison group and found no significant differences in activity levels during the most active 10 hr of the day across all groups. Additionally, future comparative studies with neurodegenerative groups such as AD would be valuable for broader validation and dissecting out the individual contributions of the varying pathologies to rest–activity rhythms. Finally, although comparable in size to the previous available studies in DLB,^28^ the modest sample size would have limited our associations to those with larger effect sizes. In view of this and the exploratory nature of the study, we did not control for multiple comparisons^13^, ^14^; however, the strength and direction of the correlations were in line with our prespecified hypotheses and support the design of future large multicenter cohorts aimed at validating these findings in disease and prodromal cohorts.

In conclusion, this study demonstrates that wrist actigraphy is well tolerated by DLB patients and that nonparametric measures provide useful insights into rest–activity disturbances, closely correlating with core neuropsychiatric and motor features.

## Author Roles

(1) Research project: A. Conception, B. Organization, C. Execution; (2) Statistical analysis: A. Design, B. Execution, C. Review and critique; (3) Manuscript: A. Writing of the first draft, B. Review and critique.

J.A.: 1B, 1C, 2B, 3A

N.K.H.C.: 1A, 1B, 1C, 2A, 3B

J.E.P.: 1A, 2A, 2B, 3B

M.C.‐S.: 3B

A.L.: 1B, 3B

R.R.G.: 3B

S.J.G.L.: 1A, 2C, 3B

E.M.: 1A, 1B, 1C, 2A, 2C, 3B

## Disclosures

**Ethical Compliance Statement:** Written informed consent was obtained from all patients, and ethical approval was obtained from the Sydney University Human Research Ethics Committee (HREC number 2013/HE000945). We confirm that we have read the journal's position on issues involved in ethical publication and affirm that this work is consistent with those guidelines.

**Funding Sources and Conflicts of Interest:** This project was supported by the National Health and Medical Research Council Emerging Leadership Fellowship (2008565) and Leadership Fellowship (1195830). United States Department of Defense Congressionally Directed Medical Research Program (PD220061). The authors declare no competing interests.

**Financial Disclosures for the Previous 12 Months:** E.M. is supported by a National Health and Medical Research Council Emerging Leadership Fellowship (2008565) and the United States Department of Defense Congressionally Directed Medical Research Program (PD220061). S.J.G.L. is supported by a National Health and Medical Research Council Leadership Fellowship (1195830) and has received research funding from the Michael J. Fox Foundation and the Australian Research Council, as well as consulting for Pharmaxis.

## Supporting information


**Table S1.** Description of parametric and nonparametric actigraphy variables.
**Table S2.** Correlations between nonparametric actigraphy and core clinical features in DLB (dementia with Lewy bodies).
**Table S3.** Correlations between nonparametric actigraphy and clinical features in PD (Parkinson's disease).

## Data Availability

The data that support the findings of this study are available from the corresponding author upon reasonable request.
