# Supplementary material for: Rest–Activity Disturbances Correlate with Core Features in Dementia with Lewy Bodies
Source: Mov Disord Clin Pract. 2025 Mar 25;12(8):1140–5. doi: 10.1002/mdc3.70052 (PMC12371458; doi:10.1002/mdc3.70052)
Supplement: Supplementary file 1 — Table S1. Description of parametric and nonparametric actigraphy variables. Table S2. Correlations between nonparametric actigraphy and core clinical features in DLB (dementia with Lewy bodies). Table S3. Correlations between nonparametric actigraphy and clinical features in PD (Parkinson's disease). [file MDC3-12-1140-s001.docx]

**Rest-activity disturbances correlate with core features in dementia with Lewy bodies**

**SUPPLEMENTARY MATERIAL**

**Supplementary Table 1.** Description of parametric and non-parametric actigraphy variables.

| **Variable** | **Definition** |
| --- | --- |
| **Parametric Actigraphy Variables** | |
| Time in Bed (TIB) | Time where subjects are attempting to sleep from the time a participant went to bed to the time they arose. Qualitatively defined by the researchers using the participant’s sleep diary and ambient light sensor.^1^ |
| Total Sleep Time (TST) | The interval (within TIB) from sleep onset to offset. Onset is defined as the timepoint of the first minute the participant was scored asleep after getting into bed.^1^ |
| Percentage of Wake Time (%WT) | The percentage of time spent awake between sleep onset to sleep offset. The percentage of wake after sleep onset, over total sleep time.^1^ |
| **Non-parametric Actigraphy Variables** | |
| Inter-daily Stability (IS) | Reflects the stability of rest-activity rhythm and the coupling of the circadian rhythm with the light cycle of the day.^2, 3^ |
| Intra-daily Variability (IV) | Reflects the fragmentation of the rest-activity rhythm.^2, 3^ |
| Average daily activity during least-active 5 hours of each day (L5) | Reflects movements such as nocturnal arousals and awakenings and may be a proxy marker for sleep disturbances, such as dream-enactment behavior.^2, 3^ |
| Average activity during the most-active 10 hours of each day (M10) | Reflects activity during the day and may be a proxy marker for daytime napping.^2, 3^ |
| Relative Amplitude (RA) | The ‘amplitude’ of circadian rhythm is represented by the standardized difference between the average activity during the most-active 10 hours of each day and the average activity during the least-active 5 hours of each day as calculated by the following equation: (M10-L5)/(M10+L5).^2, 3^ |

Abbreviations: %WT, Percentage Wake Time; IS, Inter-daily Stability; IV, Intra-daily Variability; L5, Average daily activity during least-active 5 hours of each day; M10, Average activity during the most-active 10 hours of each day; RA, Relative Amplitude; TIB, Time in Bed; TST, Total Sleep Time.

**Supplementary Table 2.** Correlations between non-parametric actigraphy and core clinical features in DLB.

|  |  | **IS** | **IV** | **M10** | **L5** | **RA** |
| --- | --- | --- | --- | --- | --- | --- |
| Disease Duration | r_s_  p | -0.206  0.545 | 0.375  0.256 | 0.264  0.433 | 0.539  0.087 | -0.530  0.093 |
| MMSE | r_s_  p | 0.207  0.594 | 0  1 | 0.390  0.300 | -0.454  0.220 | -0.218  0.572 |
| CAF Total | r_s_  p | **-0.701***  **0.016** | 0.046  0.892 | 0  1 | 0.074  0.829 | -0.149  0.662 |
| PsycH-Q – Section 1 | r_s_  p | **-0.801****  **0.009** | 0.633  0.067 | -0.430  0.248 | 0.203  0.601 | -0.559  0.117 |
| SCOPA-S (Day) | r_s_  p | **-0.659***  **0.027** | 0.219  0.517 | -0.274  0.415 | -0.009  0.979 | -0.289  0.389 |
| MDS-UPDRS-III | r_s_  p | -0.132  0.698 | 0.219  0.518 | **-0.720***  **0.013** | -0.515  0.105 | -0.066  0.846 |
| MDS-UPDRS-IV | r_s_  p | 0  1 | 0  1 | 0  1 | 0  1 | 0  1 |

** Correlation is significant at the 0.01 level (2-tailed). * Correlation is significant at the 0.05 level (2-tailed).

Abbreviations: %WT, Percentage Wake Time; CAF, the Clinician Assessment of Fluctuation Scale; DLB, Dementia with Lewy bodies; IS, Inter-daily Stability; IV, Intra-daily Variability; L5, Average daily activity during least-active 5 hours of each day; M10, Average activity during the most-active 10 hours of each day; MDS-UPDRS-III, the Movement Disorders Society Unified Parkinson’s Disease Rating Scale Section III; MDS-UPDRS-IV, the Movement Disorders Society Unified Parkinson’s Disease Rating Scale Section IV; MMSE, Mini-mental state examination the Clinician Assessment of Fluctuation Scale; PsycH-Q, the Psychosis and Hallucinations Questionnaire; RA, Relative Amplitude; SCOPA-S (Day), Outcomes in Parkinson’s Disease – Daytime Sleep; TIB, Time in Bed; TST, Total Sleep Time.

**Supplementary Table 3.** Correlations between non-parametric actigraphy and clinical features in PD.

|  |  | **IS** | **IV** | **M10** | **L5** | **RA** |
| --- | --- | --- | --- | --- | --- | --- |
| Disease Duration | r_s_  p | 0.487  0.108 | -0.030  0.917 | 0.341  0.278 | 0.105  0.746 | -0.027  0.944 |
| MMSE | r_s_  p | -0.271  0.421 | 0.281  0.403 | 0.306  0.361 | 0.551  0.078 | -0.032  0.924 |
| SCOPA-S (Day) | r_s_  p | -0.014  0.965 | -0.080  0.805 | -0.258  0.417 | -0.269  0.398 | 0.256  0.421 |
| MDS-UPDRS-III | r_s_  p | 0.028  0.931 | -0.018  0.957 | -0.175  0.585 | 0.091  0.778 | -0.206  0.520 |
| MDS-UPDRS-IV | r_s_  p | -0.134  0.677 | 0.226  0.478 | 0.108  0.740 | 0.237  0.460 | -0.246  0.441 |

** Correlation is significant at the 0.01 level (2-tailed). * Correlation is significant at the 0.05 level (2-tailed).

Abbreviations: %WT, Percentage Wake Time; IS, Inter-daily Stability; IV, Intra-daily Variability; L5, Average daily activity during least-active 5 hours of each day; M10, Average activity during the most-active 10 hours of each day; MDS-UPDRS-III, the Movement Disorders Society Unified Parkinson’s Disease Rating Scale Section III; MDS-UPDRS-IV, the Movement Disorders Society Unified Parkinson’s Disease Rating Scale Section IV; MMSE, Mini-mental state examination the Clinician Assessment of Fluctuation Scale; PD, Parkinson’s disease; RA, Relative Amplitude; SCOPA-S (Day), Outcomes in Parkinson’s Disease – Daytime Sleep; TIB, Time in Bed; TST, Total Sleep Time.

**REFERENCES**

1. Fekedulegn D, Andrew ME, Shi M, Violanti JM, Knox S, Innes KE. Actigraphy-based assessment of sleep parameters. Annals of Work Exposures and Health 2020;64(4):350-367.

2. Gao C, Haghayegh S, Wagner M, et al. Approaches for assessing circadian rest-activity patterns using actigraphy in cohort and population-based studies. Current Sleep Medicine Reports 2023;9(4):247-256.

3. Blume C, Santhi N, Schabus M. ‘nparACT’package for R: A free software tool for the non-parametric analysis of actigraphy data. MethodsX 2016;3:430-435.
